# The Impact of Human Papillomavirus Infections on Recurrent Pregnancy Loss: A Review of the Literature

**DOI:** 10.3390/diseases12090214

**Published:** 2024-09-13

**Authors:** Dimitra Dedousi, Anastasios Potiris, Athanasios Zikopoulos, Theodoros Karampitsakos, Spyridon Topis, Charikleia Skentou, Angeliki Gerede, Panagiotis Christopoulos, Athanasios Zachariou, Ekaterini Domali, Peter Drakakis, Sofoklis Stavros

**Affiliations:** 1 First Department of Obstetrics and Gynecology, Alexandra Hospital, Medical School, National and Kapodistrian University of Athens, 115 28 Athens, Greece; dimitradedousi@hotmail.com (D.D.); kdomali@yahoo.fr (E.D.); 2 Third Department of Obstetrics and Gynecology, University General Hospital “ATTIKON”, Medical School, National and Kapodistrian University of Athens, 124 62 Athens, Greece; thanzik92@gmail.com (A.Z.); theokarampitsakos@hotmail.com (T.K.); spyros.topis1996@gmail.com (S.T.); pdrakakis@med.uoa.gr (P.D.); sfstavrou@med.uoa.gr (S.S.); 3 Department of Obstetrics and Gynecology, Medical School, University of Ioannina, 451 10 Ioannina, Greece; haraskentou@uoi.gr; 4 Department of Obstetrics and Gynecology, Democritus University of Thrace, 691 00 Campus, Greece; agerede@otenet.gr; 5 Second Department of Obstetrics and Gynecology, Aretaieion University Hospital, Medical School, National and Kapodistrian University of Athens, 115 28 Athens, Greece; panchrist@med.uoa.gr; 6 Department of Urology, School of Medicine, Ioannina University, 451 10 Ioannina, Greece; zahariou@otenet.gr

**Keywords:** human papillomavirus (HPV), infection, recurrent pregnancy loss (RPL), miscarriage, pregnancy outcome

## Abstract

Human papillomavirus (HPV) infections are significantly associated with multiple adverse reproductive outcomes such as miscarriages. Pregnant women are more susceptible to an HPV infection and its prevalence increases as pregnancy progresses. In this present review, we summarize the existing evidence indicating the potential impact of an HPV infection on the occurrence of recurrent pregnancy loss (RPL). Comprehensive research of the literature was performed in the Medline/PubMed and Scopus databases. A total of 185 articles were identified and 40 full-text articles were assessed. Four studies were eligible to be included in this literature review. To our knowledge, this is the first review aiming to summarize the current state of evidence regarding the possible association of HPV infections and RPL. Recurrent pregnancy loss constitutes a distressing reproductive event and scientific research has made significant efforts to determine the causes and mechanisms that could lead to RPL. It is still unclear whether the papillomavirus infection is associated with an increased risk for recurrent miscarriages. Research in the field revealed conflicting results and their deductions are limited by methodological limitations. Given the high prevalence of HPV infections and their potential role in the occurrence of adverse outcomes during pregnancy, further research is required to clarify the possibility of an HPV infection being a potential risk factor for recurrent miscarriages.

## 1. Introduction

Human papillomavirus (HPV) is the most common sexually transmitted infection worldwide [1]. Detectable HPV infections can be identified in approximately 12% of women globally; a prevalence influenced by geographic differences, ethnicity, and age [2]. The incidence of HPV infections is most common in women under 30 years old and in those between the ages of 55 and 64 [3].

Human papillomavirus (HPV) is a small non-enveloped dsDNA virus and a member of the family Papillomaviridae, which includes more than 200 known genotypes [4]. The virus exhibits a strong affinity for the squamous epithelial cells of the uterine cervix [1]. Although most HPV infections are asymptomatic and self-resolved, persistent HPV infection remains a major concern, since it has been associated with the growth of various malignancies, including oropharyngeal, anogenital, and cervical cancers [5]. HPV types are classified into high-risk and low-risk categories based on their ability to induce cancer. HPV types 16 and 18 are the most oncogenic genotypes, leading to approximately 70% of all cervical cancers around the world. Low-risk types can cause benign lesions such as genital warts and respiratory papillomatosis [6].

Pregnant women are more vulnerable to HPV infections due to immunosuppression in pregnancy. A higher prevalence of HPV expression is observed during the second and third trimesters [7,8]. The higher rates of HPV infections during pregnancy, along with the current evidence, suggest that HPV can infect the trophoblast and replicate its genome [9]. Hence, HPV could also contribute to adverse pregnancy and perinatal outcomes, including a miscarriage, fetal growth restriction (FGR), preterm birth, low birth weight, the premature rupture of membranes (PROM), and prenatal death [5]. Recent studies have demonstrated that the E6 and E7 oncogenic genes of HPV can modify the characteristics of trophoblastic cells, such as cellular survival, the adhesion of the trophoblast to the endometrium, proliferation, and differentiation. These alterations could change the physiology of the trophoblasts and the placenta, leading to adverse obstetric complications in pregnancy [10].

Recurrent pregnancy loss (RPL) constitutes a significant reproductive health issue affecting up to 5% of couples trying to conceive [11]. The American Society for Reproductive Medicine and the European Society of Human Reproduction and Embryology define recurrent pregnancy loss as the loss of two or more consecutive pregnancies before the 24th week of pregnancy [12,13]. Several factors have been linked to recurrent miscarriages, including uterine anatomical abnormalities, metabolic and endocrine diseases, genetic or immunological disorders, thrombophilia, and environmental factors [11,14,15]. However, the causes of recurrent abortions remain unexplained in more than 50% of cases [16]. Additionally, specific infections affecting the female reproductive system, such as chlamydia, toxoplasma gondii, mycoplasma hominis, herpes simplex virus, rubella, human cytomegalovirus, and SARS-CoV-2, play a vital role in the occurrence of recurrent miscarriages [17,18]. Similarly, HPV infections have been observed in women with recurrent abortions [19]. As far as the effects of a co-infection with HPV and SARS-CoV-2 on the occurrence of recurrent pregnancy loss are concerned, the impact of having SARS-CoV-2 and HPV at the same time has not been extensively studied. However, the immune response to one virus may influence the other, potentially complicating pregnancy outcomes. Nevertheless, according to Luigi et al., the social distance caused by the COVID-19 pandemic did not reduce the transmission of HPV infections. On the contrary, it led to limited access to healthcare services, resulting in an increase in undiagnosed HPV cases [20].

The aim of this present review is to investigate the potential association between HPV infections and the occurrence of recurrent miscarriages by summarizing the findings presented in the existing published literature.

## 2. Materials and Methods

Comprehensive research of the literature was performed in the Medline/PubMed and Scopus databases with the aim of identifying potentially relevant studies and articles regarding the association between human papillomavirus (HPV) infections and recurrent pregnancy loss (RPL). The search algorithm included the following keywords: “Human Papilloma virus”, “HPV”, “HPV infection”, “Recurrent Pregnancy Loss”, “Recurrent Miscarriage”, and “Recurrent Abortion”. These search terms were either used as presented, separately, or in combination with the help of the Boolean administration (OR, AND). Neither a time limit nor other filters were applied to either database.

The inclusion criteria for this present review included studies that (1) examined the relationship between HPV infections and recurrent miscarriages, (2) were patient–control studies with adequate primary data (number of subjects per population and statistical data for a comparison of the *p*-values or q-values and/or foldchange), (3) included patients who had at least two consecutive miscarriages and controls who had at least one successful pregnancy or a history of legal termination of pregnancy without a history of miscarriage. Similarly, the exclusion criteria included studies that (1) did not relate to HPV infections or recurrent miscarriages, (2) did not provide adequate data for extraction, (3) included patients who were diagnosed with other causes of recurrent miscarriages, and (4) were written in a language other than English.

Regarding the quality assessment and risk of bias assessment of the included studies, a critical evaluation of each study’s sample (the size of the sample), methodology (the study design and compared groups), outcome presentation (the clarity and relevance of the reporting outcomes), and confounding factors (potential biases and the different methods of estimating recurrent miscarriages or HPV infections) was performed. This critical evaluation helped in the interpretation of each study’s outcomes and led to a better presentation of our results and discussion about the effect of HPV infections on recurrent pregnancy loss. A formal risk of bias and quality assessment was not performed due to the narrative nature of this review.

## 3. Results

**Figure 1:**
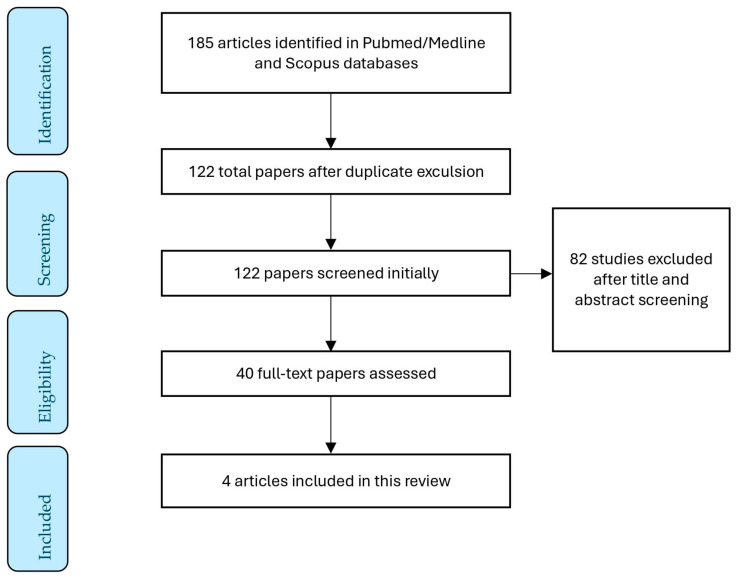
Flow diagram of the study selection process.

A total of 185 articles were identified from the databases. The initial screening of the literature was performed by the allocated reviewers (D.D and A.P.). In total, forty full-text articles were assessed, and four studies were found to be eligible to provide information for this literature review. Figure 1 schematically presents the study selection process. The following information was extracted from the included studies: year of publication, country, ethnic group, the diagnostic criteria for recurrent miscarriages, the sample size (the number of patients and controls), the mean age of the compared groups, the HPV detection method, and the tissue from which the HPV was isolated. The variable *p*-value was also extracted. Table 1 summarizes the main outcomes of the four included studies in this review.

The study conducted by Ticconi et al. [21], published in 2013, investigated the correlation between human papillomavirus (HPV) infections and recurrent abortions. In this retrospective case–control study, 524 women of childbearing age were included. The women in the study were divided into two groups. Group 1 (the cases) included 49 women clinically diagnosed to experience recurrent pregnancy loss. The participants’ average age was 38.32 years with a standard deviation of 7.12. The women with anatomic defects, genetic problems, immunological diseases, thrombophilia, and endocrine disorders were excluded from the study. Group 2 (the controls) comprised 475 healthy women who had experienced at least one full-term pregnancy and had no history of miscarriages. Their mean age was 36.47 ± 8.87 years, which did not differ statistically from that of Group 1. Two distinct methods were used in the present study for detecting HPV DNA: a Hybrid Capture II (HC2) assay and a polymerase chain reaction (PCR). The HC2 test was carried out in 93 participants constituting 17.75% of the total sample (seven women from Group 1 and 86 from Group 2), while a PCR was performed in 431 women comprising 82.25% of the total sample (forty-two women from Group 1 and 389 from Group 2). In Group 1, 26.53% (13/49) of the women tested positive for HPV DNA, compared to 61.89% (294/475) in Group 2. The difference between the groups was statistically significant (*p* < 0.001). Thus, the frequency of HPV-positive DNA tests was significantly higher in the control women than in the women with recurrent miscarriages, regardless of the method used. These findings suggest that women experiencing recurrent miscarriages may exhibit a lower susceptibility to HPV infection compared to those without any history of miscarriage who have had at least one full-term pregnancy. Furthermore, within the 30–39 years age range, the prevalence of HPV DNA positivity differed significantly, with three out of twenty women in Group 1 and 88 out of 148 women in Group 2 testing positive (*p* < 0.0005). Finally, Group 1 and Group 2 did not exhibit significant differences in the prevalence of specific HPV types or in cytological and histological findings.

Another study published in 2013 by Conde-Ferráez et al. [22] determined the possible association of an HPV infection in the uterine cervix with spontaneous and recurrent miscarriages. The scientific team performed a case–control study including a total of 281 women, corresponding to 143 cases and 138 controls. The participants were classified as the cases if they experienced a spontaneous miscarriage requiring curettage and as the controls if they were pregnant women attending for term delivery. Regarding the cases, the participants’ age ranged from 14 to 47 years, with a mean age of 28.6 years. Of the 143 cases, 20.56% (29 out of 141) reported a history of previous pregnancy loss. Two cases were missing data. Additionally, 86% of the cases (123 out of 143) were in the first trimester of gestation, while 14% (20 out of 143) were in the gestational period ranging from 13 to 20 weeks. As for the controls, the age range was 16 to 38 years (with a mean of 25.3 years). The average gestation was 39 weeks (with a range of 36 to 42). A PCR analysis was performed to detect the presence of HPV DNA in the cervical samples. Due to an internal control b-globin amplification failure, three samples were excluded from the analysis, and one patient’s cervical sample was unavailable for analysis. Consequently, 277 women participated in the HPV testing (139 cases and 138 controls). Among these participants, 55 were identified as HPV-positive, yielding a prevalence of 19.8% in the examined group. The HPV prevalence was higher among the cases, at 24.4% (34 out of 139), compared to the controls, where it was 15.2% (21 out of 138). Regarding recurrent abortions, 27.3% of HPV-positive women among the cases had at least one prior pregnancy loss, compared to 17.43% of their HPV-negative counterparts. However, the bivariate analysis indicated that this finding was not statistically significant (*p* = 0.2321961). HPV types 16 and 58 were most frequently observed in both groups. Moreover, an HPV cervical infection was significantly associated with multiple sexual partners, alcohol consumption before pregnancy, and passive tobacco exposure. It is important to mention that, in the case group, 60.2% (85/141) were found to be positive with any TORCH agent. However, this was not significantly correlated with a history of spontaneous or recurrent miscarriages. The presence of TORCH agents in the cases was not linked to recurrent abortions. Among the women positive with any TORCH infection, 22.3% (19 out of 85) also tested positive for an HPV cervical infection. To eliminate the influence of active TORCH infections, all 54 TORCH-positive cases were excluded. From the 54 TORCH-positive cases, 15 of them were positive with an HPV infection, while 39 were negative. Therefore, the results showed that a cervical HPV infection was more frequent in the cases than in the controls, although it was not significantly associated with spontaneous or recurrent miscarriages.

The case–control study by Hammad et al. in 2016 [23] investigated the potential involvement of HPV as a contributing factor to recurrent pregnancy loss. Paired samples including both placenta and urine were collected. The case group included samples from women suffering from recurrent miscarriages. The cases had an average age of 32.67 years (±3.209) and a gestational age ranging from 8 to 14 weeks (with a mean of 10.93 weeks, ±1.982). The samples in the control group were obtained from women with their first missed miscarriage, lacking a history of recurrent pregnancy loss and having previously experienced at least one uncomplicated pregnancy. After extracting genomic DNA from the collected samples, three different PCR methods were applied including conventional, nested, and real-time PCR assays for the comparative evaluation. According to the results, an HPV infection was detected in 16.6% and 26.7% of the placental samples and in 6.6% and 36.7% of the urine samples using a PCR and either a nested PCR or real-time PCR. Therefore, the results showed a significant difference between the number of miscarriages in the HPV-positive and HPV-negative placenta samples. These findings suggest that an HPV infection of trophoblasts might cause placental dysfunction and contribute to obstetric complications, including recurrent pregnancy loss. Finally, HPV-16 was predominant in both samples, followed by HPV-31.

The scientific team of Khalid et al. [10] aimed to reveal in 2023 the role of HPV-16 and its relationship to the incidence of recurrent miscarriages. A cross-sectional hospital-based study was performed, including a total of 134 Iraqi women. The participants in the study were categorized into two groups. The RPL group was represented by 114 women experiencing recurrent pregnancy loss, and their age range was 20–40 years. The control group included 20 women with a history of two or more uneventful pregnancies and no prior miscarriages. All the women who participated in the study underwent a deep vaginal swab collection, followed by HPV DNA detection using a real-time PCR and broad-spectrum HPV-specific GP5+/6+ primers. The results demonstrated that 10.53% (12 out of 114) of the women suffering from recurrent pregnancy loss were positive with HPV, while none of the control group tested positive, suggesting a strong relationship between human papillomavirus (HPV) and recurrent miscarriages. These findings are consistent with those of Hammad et al. [6], who found a greater prevalence of HPV among women experiencing recurrent abortions. Additionally, the study showed that women with five or more miscarriages exhibited the highest mean of HPV load (13.724 copy/cell), while the lowest mean (7.953 copy/cell) was recorded among the RPL women with three previous abortions. The researchers also concluded that younger women with higher levels of HPV DNA tended to experience a higher number of miscarriages.

## 4. Discussion

To our knowledge, this is the first review aiming to summarize the current state of evidence regarding the possible association of human papillomavirus (HPV) infections and recurrent pregnancy loss (RPL). Scientific research has made significant effort to determine the causes and mechanisms that could lead to recurrent miscarriages. In recent years, particular interest has been given to specific infections, including HPV infection, as potential causative factors of idiopathic recurrent abortions. Human papillomavirus is among the most common sexually transmitted viral infections affecting both males and females of reproductive age [24,25]. Recent evidence suggests that these viruses can have detrimental effects on pregnancy, such as miscarriage, stillbirth, and preterm delivery [17,18,26].

According to Khalid et al., a papillomavirus infection is highly related to recurrent miscarriages. The study revealed that 10.53% of women with recurrent pregnancy loss tested positive by a PCR, whereas none of the control women exhibited positive PCR results. The squamous epithelium of the cervix can be infected by HPV and these infections may occur as early as the first trimester of pregnancy [10]. Additionally, the scientific team demonstrated that the mean HPV DNA load was significantly higher in women with a higher number of recurrent miscarriages, specifically five or more. The number of HPV DNA copies found in the RPL women who tested positive aligns with the results of previous studies that determined the number of HPV copies per cell using a qPCR [27]. The researchers also concluded that younger women with higher levels of HPV DNA tended to experience an increased number of miscarriages. However, the smaller number of women included in the control group compared to those in the RPL group could represent a limitation of the study.

The findings of the above study are also supported by Hammad et al. who presented that women experiencing recurrent abortions had a higher prevalence of HPV than controls [23]. Their results revealed a significant difference in miscarriage rates between HPV-positive and HPV-negative placenta samples, indicating a potential link between an HPV infection of the trophoblasts and adverse pregnancy outcomes. Another scientific team by Gomez et al. confirmed these effects, demonstrating that human trophoblasts have HPV receptors and promote HPV DNA replication [28]. Furthermore, You et al. concluded through studies conducted on trophoblast cell lines that an HPV infection leads to a reduction in the number of trophoblasts, since the introduction of E6 and E7 oncoproteins promotes apoptosis in trophoblasts [29,30]. The researchers also suggested that an HPV infection of trophoblasts disrupts their adhesion to endometrium, which could result in embryo expulsion [29].

The study conducted by Conde-Ferráez et al. revealed that a cervical HPV infection was more common in the cases than in the controls, but it was not significantly linked to spontaneous or recurrent miscarriages [22]. However, the results of this study could be limited by the small sample size. Additionally, it is important to mention that, in this particular study, TORCH infections were very frequent in the cases, although they were not associated with recurrent miscarriages. Nevertheless, the research team excluded all TORCH-positive cases to eliminate the potential impact of the active TORCH infections. TORCH infections are known to increase the risk of pregnancy loss and obstetric complications, attributed to their ability to induce trophoblast apoptosis and inflammatory reactions [22,31].

Although the previous studies proposed that HPV infections are more frequent among women with recurrent miscarriages, Ticconi et al. suggested that the prevalence rates of a positive HPV DNA test were significantly lower in women experiencing recurrent miscarriages than in healthy women [21]. These major findings suggest that women with recurrent miscarriages exhibit a stronger immune response. However, at the same time, this elevated immunological reaction makes them less susceptible to an HPV infection compared to women who have uncomplicated pregnancies without pregnancy loss. Furthermore, it is possible that women with recurrent abortions could have overcome previous HPV infections more rapidly than healthy women. Nevertheless, additional research is necessary to confirm these hypotheses. However, a possible limitation of this study is that two distinct methods were used for detecting the HPV DNA, including a Hybrid Capture II (HC2) assay and a polymerase chain reaction (PCR). However, both methods are efficient in achieving this goal.

Overall, the current scientific evidence presents conflicting results regarding the correlation between HPV infections and the occurrence of recurrent miscarriages. The variation in HPV prevalence can be attributed to differences in HPV detection techniques, multiple risk factors such as maternal and gestational ages, the presence of genital warts, a history of cervical dysplasia, and other sexually transmitted infections [32]. Due to the existing limitations in the studies included in this review, the association between HPV infections and recurrent abortions can be regarded as possible but not undeniable. Undoubtedly, HPV in recurrent miscarriages and other adverse pregnancy outcomes requires further study. Finally, it is worth mentioning that the study conducted by Dousti et al. demonstrated that a vaccination against HPV during pregnancy does not significantly increase the risk of a miscarriage [33]. Although, there is not enough evidence to indicate that an HPV vaccination is unsafe during pregnancy, it is recommended to delay the vaccination to the postpartum period [34,35].

## 5. Conclusions

In conclusion, since the results of the studies are conflicting, the potential role of human papillomavirus in recurrent pregnancy loss is intriguing and still undetermined. However, recent evidence suggests that HPV has the potential to infect the fetus and the gestational tissues, having some role to play in the occurrence of recurrent miscarriages. Further studies in this specific scientific field are recommended, as the identification of an HPV infection upon an RPL diagnosis can be of the utmost importance in pre-conception genetic counseling for achieving successful pregnancies in the future.

## Figures and Tables

**Table 1:** Main characteristics and outcomes of the included studies in this review.

Study	Study Design	Sample	HPV Detection	Main Outcomes
Ticconi et al., 2013 [21]	Retrospective case–control study	Cervical samples	Hybrid Capture II (HC2) assay Polymerase chain reaction (PCR)	The prevalence of HPV-positive DNA tests was lower in women with recurrent miscarriages compared to controls.
Conde-Ferráez et al., 2013 [22]	Case–control study	Cervical samples	Polymerase chain reaction (PCR)	Cervical HPV infections were more frequent in women experiencing miscarriage than in controls. However, there was no significant correlation with spontaneous or recurrent miscarriages.
Hammad et al., 2016 [23]	Case–control study	Placenta and urine samples	Conventional PCR Nested PCR Real-time PCR	The prevalence of HPV-positive DNA tests was higher in women with recurrent miscarriages compared to controls.
Khalid et al., 2023 [10]	Cross-sectional study	Vaginal swabs	Real-time PCR	Human papillomavirus is highly related to recurrent pregnancy loss.

## Data Availability

No new data were created or analyzed in this study. Data sharing is not applicable to this article.
